# Differential Jasmonate Profiles in Oat Roots and Leaves Reveal a Role for 12-Oxo Phytodienoic Acid (OPDA) in Drought Tolerance by Modulating Root Growth

**DOI:** 10.3390/plants15091312

**Published:** 2026-04-24

**Authors:** Francisco J. Canales, Gracia Montilla-Bascón, Nicolas Rispail, Vicent Arbona, Luis A. J. Mur, Elena Prats

**Affiliations:** 1 Department of Life Sciences, Aberystwyth University, Aberystwyth SY23 3FL, UK; lum@aber.ac.uk; 2 Institute for Sustainable Agriculture, Spanish National Research Council (CSIC), 14004 Córdoba, Spain; gmontilla@ias.csic.es (G.M.-B.); nrispail@ias.csic.es (N.R.); elena.prats@ias.csic.es (E.P.); 3 Department de Biologia, Bioquímica i Ciències Naturals, Universitat Jaume I, Campus del Riu Sec, 12006 Castelló de la Plana, Spain; arbona@uji.es

**Keywords:** jasmonates, oat, OPDA, drought tolerance, root growth, hormonal signaling, abiotic stress

## Abstract

Jasmonates (JAs) are a diverse group of jasmonic acid (JA)-linked metabolites, including the biosynthetic intermediate 12-oxophytodienoic acid (OPDA). Although changes in JAs have been associated with plant responses to abiotic stress, the involvement and kinetics of specific forms such as JA, JA-Ile and OPDA require further clarification. This study analyzed jasmonate profiles in roots and leaves of two oat genotypes differing in drought tolerance. Jasmonates were quantified using UPLC-MS/MS, expression of key biosynthetic genes was assessed by qRT-PCR, and JA/OPDA treatments were applied to evaluate their effects on physiological and morphological responses to drought. Drought induced contrasting jasmonate dynamics in roots and leaves, with overall JA levels increasing in leaves and decreasing in roots, with genotype- and compound-specific differences. JA and JA-Ile ((+)-7-iso-jasmonoyl-L-isoleucine) showed similar trends, whereas OPDA displayed a distinct pattern. The tolerant genotype exhibited an early and marked reduction in root OPDA, while the susceptible one showed minimal change. Exogenous OPDA increased drought symptoms, reduced leaf relative water content and strongly decreased root length by limiting the formation of new thin roots. In contrast, JA application alleviated drought symptoms, reflected in a lower area under the drought progress curve, without affecting root length. Results suggest that under water deficit, reduced OPDA, likely due to its conversion into JA and JA-Ile, is associated with the development of small-diameter roots essential for maintaining water status in oat. Together, these results highlight tissue-specific differences in jasmonate dynamics during drought and show that OPDA and JA treatments lead to distinct drought-related responses in both leaves and roots.

## 1. Introduction

**Figure 1:**
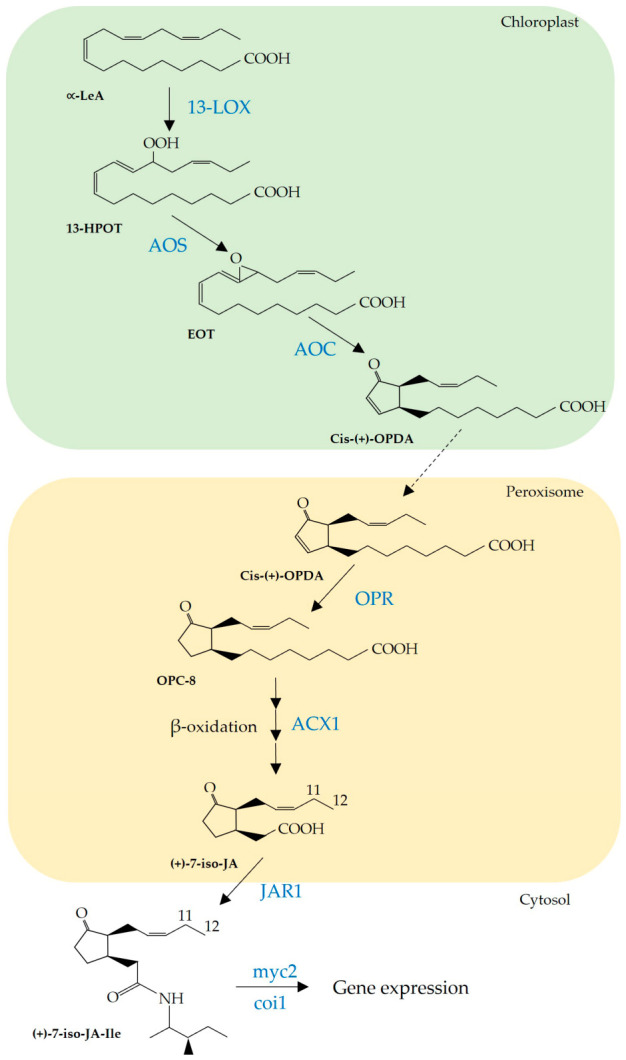
Simplified jasmonic acid (JA)/(JA-Ile) biosynthetic pathway from ∝-linolenic acid. Enzymes are written in blue. In the 13-LOX pathway, the product (13S)-hydroperoxy-octadecatrienoic acid (13-HPOT), is a substrate for allene oxide synthase (AOS). AOS converts 13-HPOT to (12,13S)-epoxyoctade catrienoic acid (EOT). Allene oxide cyclase (AOC) converts EOT to cis-(+)-12-oxo-phytodienoic acid (12-OPDA). OPDA is then transported into peroxisome where it is converted to jasmonic acid (JA) by several rounds of β-oxidation. Jasmonic acid moves to the plant cytoplasm where it can be converted to (+)-7-iso-jasmonoyl-L-isoleucine (JA-Ile) by JAR1. Coronatine insensitive1 (COI1) and bHLHzip transcription factor (MYC2) control additive subsets of JA-dependent responses.

Jasmonic acid (JA), its conjugate jasmonoyl-isoleucine (JA-Ile) (commonly designated as jasmonates; JAs) and intermediates of the JA biosynthetic pathway such as 12-oxo-phytodienoic acid (OPDA) play a crucial role in regulating developmental processes and plant responses to biotic stresses [1,2]. JAs and their intermediates are synthesized from polyunsaturated fatty acids released by plastid lipases via the octadecanoid pathway. The initial step is catalyzed by 13-lipoxygenases (LOX), which oxygenate linolenic (18:3n-3) fatty acids to form hydroxyperoxy derivatives. These derivatives are subsequently converted by allene oxide synthase (AOS), into an unstable allene oxide intermediate, which is rapidly and stereospecifically cyclized to form cis-(+)-12-oxo-phytodienoic acid (OPDA) by allene oxide cyclase (AOC) [3,4]. Following transport to the peroxisome, OPDA is reduced by OPDA reductase3 (OPR3) and then activated and shortened via three rounds of β-oxidation to yield (+)-7-iso-jasmonic acid (JA), which epimerizes to the more stable trans isomer (-) JA [2]. Subsequently, JA may be then transformed in its isoleucine (Ile) conjugated (JA-Ile) in a reaction catalyzed by the amino acid conjugate synthetase JASMONATE RESISTANT1 (JAR1) ([5]; Figure 1).

Jasmonic acid (JA) was originally identified as a key regulator of plant responses to biotic stresses, particularly defence against necrotrophic pathogens and insect herbivores, and it is now recognized as a central component of plant immune signalling. At the mechanistic level, JA coordinates multiple layers of the defence response by activating the COI1–JAZ–MYC transcriptional module, which controls the expression of genes encoding defence proteins, secondary-metabolite biosynthetic enzymes, and antimicrobial compounds. In cereals, JA-mediated defences integrate pattern-triggered and effector-triggered immunity, modulate reactive oxygen species and calcium signalling, and interact with other hormonal pathways, including salicylic acid and ethylene, to fine-tune resistance outcomes depending on pathogen lifestyle and environmental context [6].

In addition to their well-established roles in defence, jasmonates influence developmental processes, including root architecture, which might in turn influence nutrition acquisition and adaptation to abiotic stresses such as drought [7,8]. Elevated levels of JA and JA-Ile typically inhibit primary root elongation by reducing cell division and expansion in the root meristem, acting through the MYC2–JAZ signalling module [9]. These effects integrate with auxin and ethylene pathways, contributing to the fine modulation of root system architecture under changing environmental conditions [10,11]. In cereals, jasmonate-related mechanisms have been linked to root system modulation and drought responses. In wheat, studies manipulating the activity of 12-oxophytodienoate reductases (OPRIII) have shown that altering the conversion of OPDA to downstream jasmonates affects root architecture, with loss-of-function alleles leading to increased OPDA accumulation and longer seminal roots, and higher OPRIII dosage reducing root elongation and enhancing JA/JA-Ile levels [12]. Similarly, jasmonates have been implicated in the regulation of seminal root formation under stress conditions in wheat [13]. Despite these advances in monocot models, information on jasmonate-mediated regulation of root development or drought adaptation in cereals, and particularly in oat, remains scarce.

While JA has long been regarded as the sole biologically active end product of the octadecanoid (“oxylipin”) pathway, accumulating evidences indicates that other oxylipins, particularly OPDA, exhibit distinct biological activities. For example, while JA and JA-Ile are essential for many defence responses, certain processes such as embryo development [14] and seed germination [15] specifically require OPDA. Moreover, OPDA appears to be more effective than JA in promoting mechano-transduction responses [16]. Analyses of JA-deficient mutants in *Arabidopsis* revealed that OPDA contributes to resistance against pathogens and herbivores [17] and in soybean, OPDA induces higher levels of phytoalexins compared to JA [18]. Furthermore, transcriptional analysis in both wild-type and MpCOI1 mutant plants, lacking JA-Ile, treated with OPDA have identified COI1-independent regulatory networks governing thermotolerance-related genes, suggesting that OPDA plays a protective role against heat stress in *Marchantia* through its electrophilic properties [19]. Importantly, OPDA is not recognized by the COI1-JAZ co-receptor complex [20], which mediates the ubiquitin-dependent degradation of JAZ repressors to activate JA-dependent gene expression. These findings collectively underscore that OPDA and JA/JA-Ile may act via distinct signalling pathways and fulfil unique physiological roles. Furthermore, several jasmonate-mediated processes occur independently of JAR1. For example, anthocyanin accumulation, which contributes to stress protection, and wound responses have been observed even when JAR1 activity is compromised [21,22].

In *Arabidopsis*, this apparent independence is partly attributed to the function of AtGH3.10, which acts in a partially redundant manner with AtJAR1 during flower development and in response to wounding [23]. This redundancy highlights the complexity of jasmonic acid signalling, where multiple enzymes contribute to the conjugation of JA, ensuring a robust and flexible response to various physiological cues. Furthermore, several studies have addressed the controversy surrounding OPDA’s biological activity by analyzing complete loss-of-function alleles for OPR3. The *Arabidopsis* mutant, *opr3-1*, was JA-deficient yet accumulated OPDA upon wounding [24] and has since then been used to distinguish between OPDA- and JA-specific responses [25]. More recently, the identification of *opr3-3*, with a complete loss of OPR3 activity [26], confirmed the existence of an alternative, OPR3-independent pathway for JAs biosynthesis and suggested that OPDA itself may function as a signalling molecule [2].

In addition to their roles in plant development and defence responses, jasmonates have also been associated with plant tolerance to several abiotic stresses, including heavy metals, temperature fluctuations, drought and salinity [27,28,29]. However, whilst some studies report that JAs improve drought tolerance, others have noted a detrimental effect on growth and yield [30], leading to an ongoing debate regarding their precise role under water-limited conditions [31]. These apparently paradoxical effects may result from differences in drought intensity and duration, tissue specificity, plant developmental stage and JA concentration [30]. Moreover, most previous studies have focused on aerial tissues, with relatively few investigations considering the functions of JAs in roots [27,32]. Jasmonates, including JA and its precursors, play a key role in regulating root development and hydraulic function. In general, increased levels of jasmonates are known to inhibit primary root elongation by suppressing cell division and expansion in the root meristem. This inhibition is mediated through the MYC2-JAZ signalling module and involves extensive crosstalk with auxin and ethylene pathways, enabling a fine-tuned modulation of root system architecture in response to environmental stresses [13,33]. Moreover, jasmonates have been implicated in the regulation of root hydraulic conductivity under stress conditions. While some studies report a protective role through aquaporin modulation, elevated jasmonate levels are generally associated with reduced root growth, which may negatively impact water uptake efficiency [13,34]. Therefore, further research is needed to elucidate the role of different JAs species in economically important crop plants.

In this work, we investigated the role of JAs during drought stress in both leaves and roots of two oat cultivars previously characterized as tolerant and susceptible to drought [35,36]. We quantified JA, JA-Ile and OPDA levels and analyzed changes in the expression of key genes in the jasmonate biosynthetic pathway during gradual water depletion. In addition, we explored the roles of different jasmonates through the exogenous application of JA and OPDA. Our results suggest discrete roles for root JAs during drought stress responses, with OPDA significantly associated with the production of fine roots to aid drought tolerance.

## 2. Results

### 2.1. OPDA Follows a Different Accumulation Pattern than JA or JA-Ile in Leaves and Roots of Resistant and Susceptible Oat Genotypes During Drought Stress

**Figure 2:**
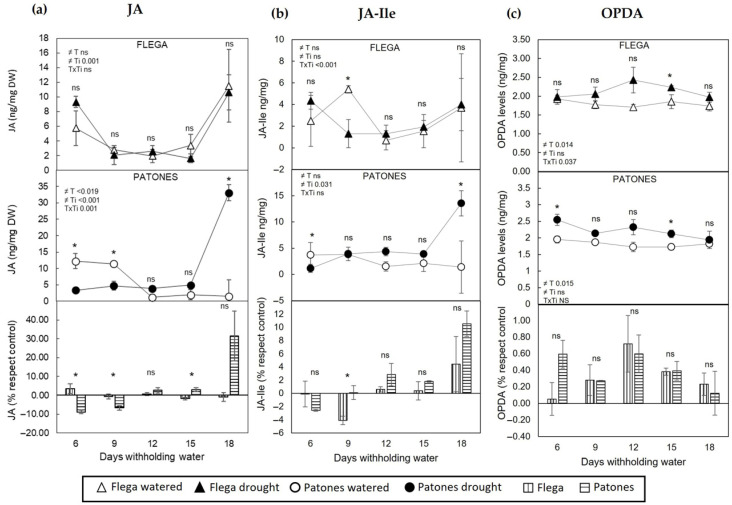
Jasmonic, Jasmonic-Ile and 12-Oxo Phytodienoic acid content in oat leaves during a drought time course (6, 9, 12, 15, 18 days withholding water). (**a**) Jasmonic, (**b**) Jasmonic-Ile and (**c**) 12-Oxo Phytodienoic acid (OPDA) were quantified in leaves of the susceptible Flega (triangles) and tolerant Patones (circles) during a drought time course of water stress (solid symbols) as compared to well-watered plants (open symbols). Lower bar panels present values expressed relative to each genotype’s own well-watered control to allow comparison of drought-induced changes independently of basal differences between genotypes (Flega, vertical lines; Patones, horizontal lines). Each set comprised at least six biological replicates (different plants) per oat genotype and treatment. Sampled tissues from 2 different plants (2 technical replicates) were pooled, making one biological replicate, so data in the figure are mean of the three biological replicates ± standard errors. * indicates significant differences between genotypes at *p* < 0.05 according to Scheffé’s post hoc test. ns indicates no significant differences. Inserts indicate overall differences between treatments (T), times (Ti) and its interaction (TxTi), as determined by ANOVA.

**Figure 3:**
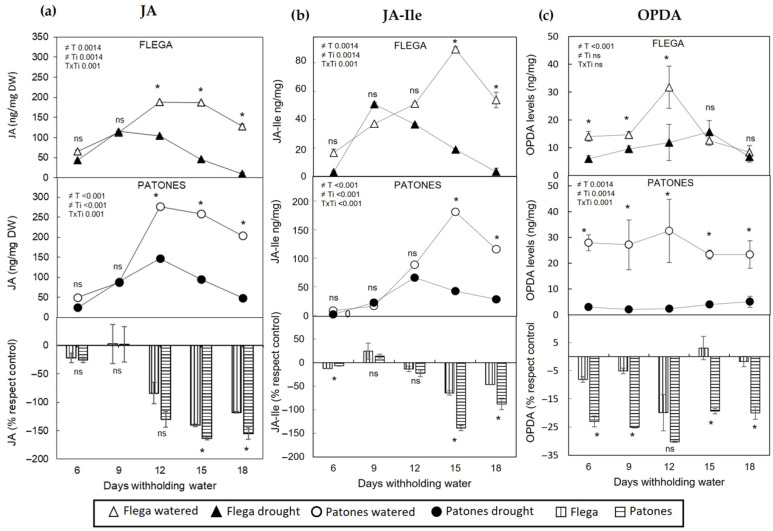
Jasmonic, Jasmonic-Ile and 12-Oxo Phytodienoic acid content in oat roots during a drought time course. (**a**) Jasmonic, (**b**) Jasmonic-Ile and (**c**) 12-Oxo Phytodienoic acid (OPDA) were quantified in roots of the susceptible Flega (triangles) and tolerant Patones (circles) during a drought time course of water stress (solid symbols) as compared to well-watered plants (open symbols). Lower bar panels present values expressed relative to each genotype’s own well-watered control to allow comparison of drought-induced changes independently of basal differences between genotypes (Flega, vertical lines; Patones, horizontal lines). Each set comprised at least six biological replicates (different plants) per oat genotype and treatment. Sampled tissues from 2 different plants (2 technical replicates) were pooled making one biological replicate, so data in the figure are mean of the three biological replicates ± standard errors. * indicates significant differences between genotypes at *p* < 0.05 according to Scheffé’s post hoc test. ns indicates no significant differences. Inserts indicate overall differences between treatments (T), times (Ti) and its interaction (TxTi), as determined by ANOVA.

Following the imposition of water stress, a differential accumulation pattern of various jasmonates (JAs) was observed in both leaves (Figure 2) and roots (Figure 3) with JA and JA-Ile displaying similar behaviour, whereas OPDA followed a distinct trend. These responses were genotype-dependent and varied according to the specific JA analyzed. Overall, there were no significant changes in JA or JA-Ile between control and droughted leaves of Flega, although a slight decrease occurred at intermediate time points followed by recovery at later stages (Figure 2a,b). However, both metabolites increased in the leaves of the tolerant genotype, Patones, at the last stage of the drought time course (Figure 2a,b). By contrast, the leaves of both genotypes showed a small but significant increase in OPDA when considering the overall drought time course (*p* = 0.014 and *p* = 0.015 for Flega and Patones, respectively; Figure 2c). When expressed relative to their respective well-watered controls, this OPDA increase was comparable between the two genotypes, as shown in the lower panel of Figure 2c. These results are consistent with our previous observations of JA dynamics in oat leaves under drought [35].

In roots, the accumulation patterns of JA and JA-Ile were also similar, differing from that of OPDA, with pronounced genotype-specific differences (Figure 3). In control roots, JA and JA-Ile levels showed a moderate increase at intermediate time points, followed by a gradual decline, although they did not return to initial values. Roots subjected to drought stress exhibited a similar temporal pattern, but with consistently lower hormone levels throughout the time course, particularly as drought stress intensified (12–18 daww, from 30 to 35% sRWC; Figure 3a,b).

This overall trend was observed in both genotypes. However, Patones showed a significantly stronger reduction in JA and JA-Ile than Flega at the latest stages of the drought time course (Figure 3a,b bottom panel). In contrast to JA and JA-Ile, OPDA accumulation in drought-stressed roots displayed a distinct genotype-dependent pattern. In drought-tolerant Patones plants, OPDA levels remained significantly suppressed throughout the entire drought period (*p* = 0.0014) relative to well-watered plants. However, susceptible Flega plants exhibited only a slight decrease in OPDA at the beginning of water stress followed by a recovery from 15 daww, reaching levels similar to those of well-watered controls by the final sampling time (Figure 3c). Consequently, OPDA was significantly lower in tolerant Patones compared to susceptible Flega throughout the whole drought time-course (Figure 3c, bottom panel).

### 2.2. Differential Expression of the Jasmonic Acid Metabolic Pathway Genes Correlates with Modulated JA, JA-Ile and OPDA Content in Roots

**Figure 4:**
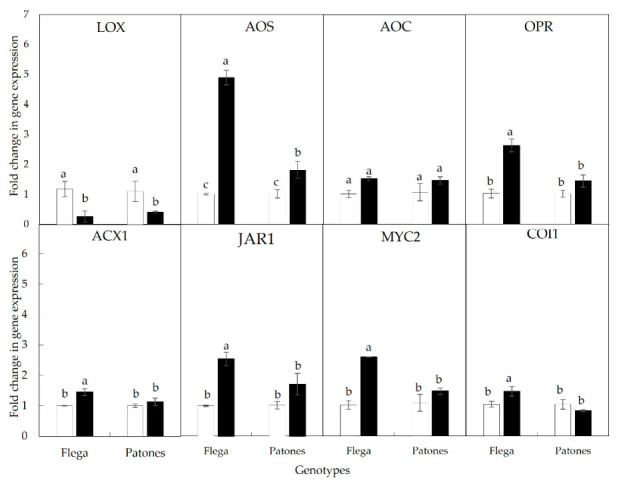
Expression of several genes involved in the Jasmonate pathway in oat roots. Expression of 13-lipoxygenase (LOX), allene oxide synthase (AOS), allene oxide cyclase (AOC), OPDA reductase3 (OPR3), acyl-CoA-oxidase 1 (ACX1), JA-aminoacid synthetase (JAR1), and bHLHzip transcription factor MYC2 (MYC2) were measured under well-watered condition (white bars) and at 20–25% soil relative water content (black bars) in Flega and Patones roots. Data, expressed as fold change in expression with respect to WT well-watered plants, are a mean of at least 3 independent biological plus 2 technical replications ± standard error. Different letters indicate significant differences for each panel at *p* < 0.05 according to Scheffé’s post hoc test.

The significant differences observed between the drought-stressed roots of the susceptible and tolerant genotype prompted us to undertake further gene expression analyses of the JA metabolic pathway (Figure 1). Quantitative RT-PCR analyses revealed that under drought conditions, LOX expression was significantly downregulated in both genotypes relative to well-watered controls (Figure 4). By contrast, AOS expression was strongly induced by drought, with Flega roots showing more than a five-fold increase compared to their well-watered counterparts (Figure 4). Notably, despite this pronounced induction of AOS, AOC expression remained unchanged in both genotypes, while several downstream genes, such as OPR3, ACX1, JAR1, the bHLH Zip transcription factor MYC (MYC2) and COI1, were significantly upregulated under drought conditions in the susceptible genotype Flega (Figure 4). The higher induction of AOS expression relative to the stable expression of AOC in Flega might therefore generate an enzymatic imbalance, potentially favouring OPDA accumulation in this genotype compared with the tolerant Patones, as reflected in the hormone profiles shown in Figure 3.

On the other hand, the reduction in OPDA levels observed in Patones under drought relative to well-watered conditions may be linked to the marked downregulation of LOX expression, which would limit the availability of the initial substrate for JA biosynthesis. Together with a more balanced regulation of AOS and AOC, this transcriptional pattern could restrict the accumulation of the allene oxide intermediate, thereby preventing OPDA build-up in the tolerant genotype.

### 2.3. Exogenous Application of OPDA but No JA Exacerbates Drought Symptoms Reducing Leaf Relative Water Content by Modulating Root Morphological Traits

**Figure 5:**
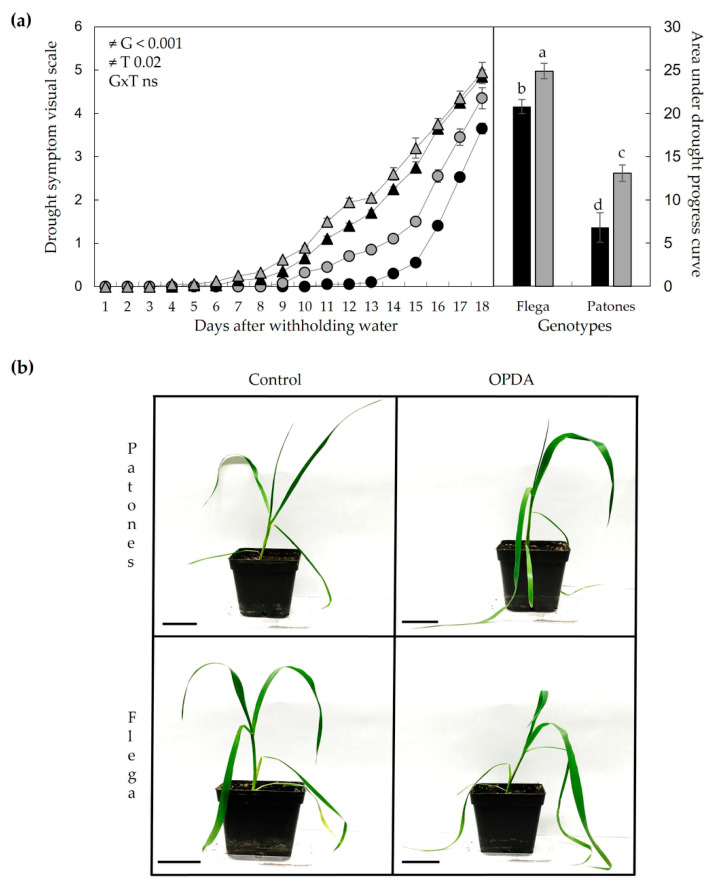
Effect of OPDA on drought symptoms in oat plants under water stress. (**a**) Drought symptoms were evaluated using a visual scale [38] after withholding water in Flega (triangles) and Patones (circles) plants. Black symbols represent drought-stressed plants, mock-treated (ethanol), while grey symbols indicate drought-stressed plants treated with OPDA. The right panel shows the area under the drought progress curve to quantify symptom differences [39]. Data represent the mean ± standard error of ten replicates. Different letters indicate significant differences at *p* < 0.05 according to Scheffé’s post hoc test. Inserts indicate interactions between genotypes (G) and OPDA treatment (T), as determined by ANOVA. (**b**) Representative images of Flega and Patones plants treated with OPDA at 20–25% sRWC. Scale bar = 5 cm.

To assess whether the pronounced reduction in OPDA observed in Patones roots under drought could contribute to its tolerance phenotype, we assessed the effects of exogenous OPDA application. OPDA was injected into the upper part of the root system during drought treatment, and its impact on drought symptom development was monitored. In both Flega and Patones, OPDA treatment did not alter the soil RWC curve, confirming that both genotypes experienced comparable levels of water stress throughout the experiment (Figure S1). Visual assessment of drought symptoms revealed significant differences between genotypes along the drought time course (*p* < 0.001) and between mock-treated controls and OPDA-treated plants (*p* = 0.02). As expected from previous studies [37], Patones exhibited milder drought symptoms, as indicated by the visual symptoms (Figure 5a,b) and the significant lower area under the drought progress curve (Figure 5a). However, OPDA treatment significantly exacerbated drought symptoms in both genotypes, leading to a marked increase in the area under the drought progress curve, with a more pronounced effect in Patones (Figure 5a). These results are consistent with the more rapid and severe turgor loss observed in the leaves after OPDA treatment (Figure 5b).

**Figure 6:**
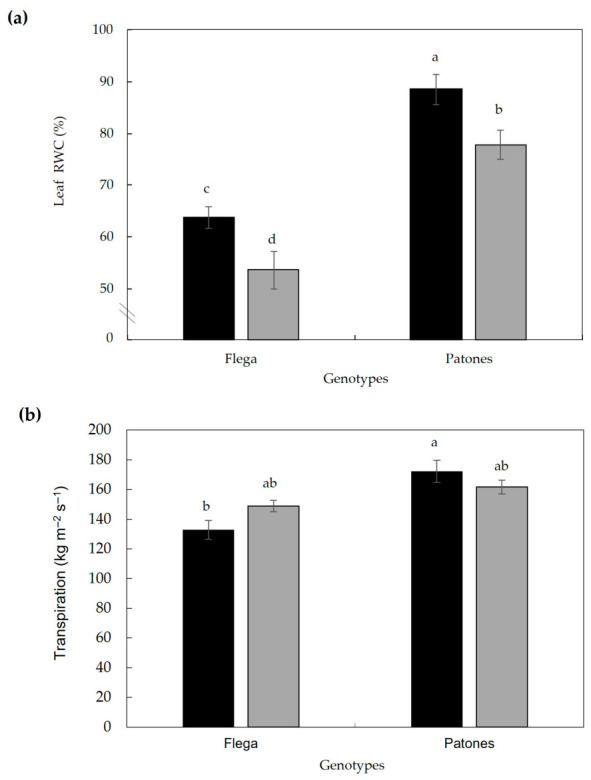
Effect of OPDA on (**a**) leaf RWC and (**b**) transpiration of oat plants exposed to water stress at 18 daww (15–20% sRWC). Black bars = mock-treated (ethanol) plants; grey bars = OPDA-treated plants. Data are mean of ten in (**a**) and five in (**b**) replicates ± standard error, respectively. Different letters indicate significant differences at *p* < 0.05 according to Scheffé’s post hoc test.

To confirm the visually observed loss of turgor in the OPDA-treated plants, leaf RWC was measured. Under drought conditions, mock-treated Patones plants maintained significantly higher leaf RWC than Flega, supporting their contrasting drought tolerance. However, OPDA treatment significantly reduced leaf RWC in both genotypes (Figure 6a). Importantly, this reduction in RWC was not accompanied by increased transpiration rates (Figure 6b), suggesting that OPDA-induced declines in leaf water status were not driven by enhanced water loss through transpiration.

**Figure 7:**
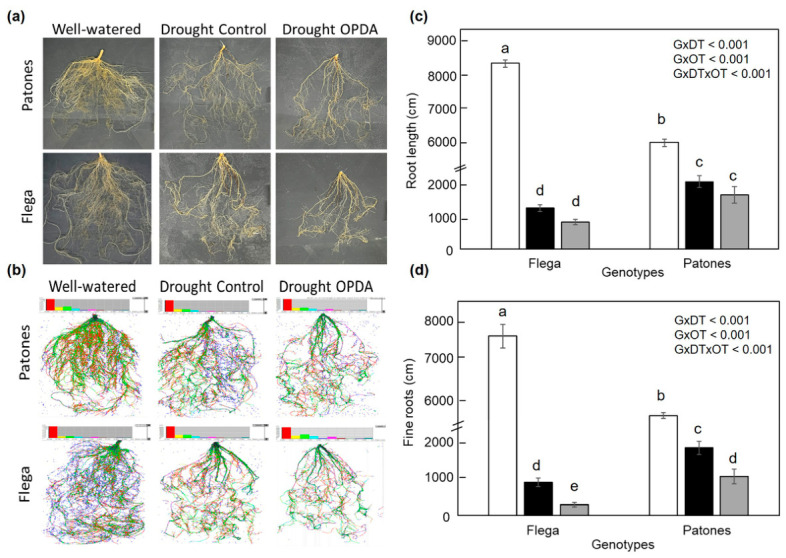
Effect of OPDA on root morphological and architectural traits at 18 daww (15–20% sRWC). (**a**) Representative pictures, (**b**) scanned images, and quantification of (**c**) total root length and (**d**) fine roots based on image analysis from plants grown under well-watered conditions (white bars), drought-stressed control (mock-treated with ethanol) (black bars), and drought-stressed plants supplemented with OPDA (grey bars). Drought-stressed control plants received the same ethanol treatment as OPDA-treated plants but without OPDA. Data represent the mean ± standard error of five replicates. Different letters indicate significant differences at *p* < 0.05 according to Scheffé’s post hoc test. Inserts indicate interactions between genotypes (G), drought treatment (DT), and OPDA treatment (OT), as determined by ANOVA.

Given that reduced RWC might result from impaired root-mediated water uptake, we next investigated whether the exogenous OPDA application affected root growth. Root morphological traits were assessed in Flega and Patones plants under well-watered and mock/OPDA-supplemented drought stress conditions (Figure 7). Flega plants exhibited a longer root system under well-watered conditions compared to Patones, which was observed visually and following scanning of the roots (Figure 7a,b). Under drought conditions, both genotypes showed a significant decrease in total and fine root length. Nevertheless, Patones exhibited a significantly smaller reduction in root length than Flega, both in terms of total root length and fine roots, with significant differences between genotypes. This suggests that Patones is less prone to drought-induced root growth inhibition. These observations are consistent with previous studies demonstrating that Patones develops a more extensive and finer root system under progressive soil drying compared with Flega, based on detailed time-course analyses in pots, rhizotrons, and semi-field conditions [37,40]. In the present study, analysis focused on the critical time point at which drought symptoms were most pronounced and root traits were most informative for discriminating genotypic responses. Two-way ANOVA on the root trait data, confirmed a significant genotype-by-treatment interaction (*p* < 0.001), supporting the differential response of the two genotypes to drought. Following OPDA application, root growth was impaired in both genotypes (Figure 7a–d). Although total root length showed only a modest, not significant reduction (approximately 12%) relative to drought-stressed controls, OPDA caused a stronger and significant reduction in the length of finest roots, with diameters under 0.5 mm, resulting in a decrease of approximately 20% in both genotypes compared with mock-treated droughted plants (Figure 7d). This indicated that OPDA preferentially affects the fine-root fraction, the most absorptive component of the root system.

To confirm the effectiveness of the treatment application via injection at the plant neck in reaching both leaf and root tissues, we measured jasmonates in these tissues 3 days after treatment. OPDA-treated plants exhibited increased levels of JA and JA-Ile relative to mock-treated controls in both roots and leaves, with a more pronounced accumulation in leaves of both Patones and Flega (Figure S2). However, increased OPDA itself was not detected at this time point, suggesting its rapid conversion to downstream JAs under drought conditions. This rapid turnover aligns with the observed reduction in endogenous OPDA levels detected during drought stress (Figure 3) and supports the notion that OPDA homeostasis is tightly regulated. Furthermore, analysis of additional hormonal responses revealed no significant changes in salicylic acid or abscisic acid levels following OPDA treatment (Figure S2). This indicates that the observed effects were specifically linked to jasmonate metabolism rather than a broader hormonal imbalance.

**Figure 8:**
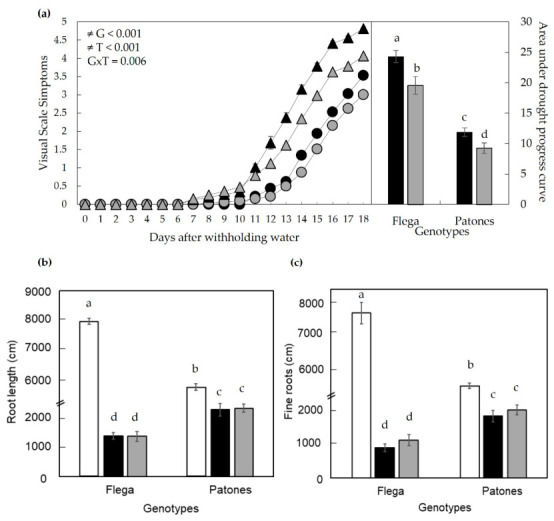
Effect of JA on drought symptoms in oat plants under water stress. (**a**) Drought symptoms were evaluated using a visual scale [38] after withholding water in Flega (triangles) and Patones (circles) plants. Black symbols represent drought-stressed plants, mock-treated (ethanol), while grey symbols indicate drought-stressed plants treated with JA. The right panel shows the area under the drought progress curve to quantify symptom differences [39]. (**b**) Total root length and (**c**) fine roots were quantified using image analysis in plants grown under well-watered conditions (white bars), drought-stressed control (mock-treated with ethanol) (black bars), and drought stress supplemented with JA (grey bars) at 18 days after withholding water (daww) (15–20% sRWC). Drought-stressed control plants received the same ethanol treatment as JA-treated plants but without JA. Data represent the mean ± standard error of ten replicates. Different letters indicate significant differences at *p* < 0.05 according to Scheffé’s post hoc test. Inserts indicate interactions between genotypes (G) and JA treatment (T), as determined by ANOVA.

To further confirm whether the increased drought symptoms observed following OPDA application were specific to OPDA rather than due to its conversion to JA, we performed a parallel experiment applying JA to a separate set of plants under similar conditions. JA application produced a response pattern clearly different from that observed following OPDA application, leading to reduced visual drought symptoms as evidenced by a lower area under the drought progress curve (Figure 8a). Unlike OPDA-treated plants, JA treatment did not lead to significant changes in the overall root length or in the length of fine roots (Figure 8b,c), compared with mock-treated drought-stressed plants. These findings indicate that the pronounced effects observed on drought symptoms and root parameters were more evident following OPDA treatment, suggesting that OPDA and JA may contribute differently to drought responses. In particular, OPDA appeared to have a large impact on the fine-root component under water deficit. Although OPDA application resulted in higher JA and JA-Ile levels at 3 days (Figure S2), the absence of JA-induced effects on root traits or drought symptoms under the same treatment conditions suggests that OPDA contributes to these responses in a manner not fully explained by downstream JA signalling.

## 3. Discussion

Over the last decade, research has highlighted an important role for jasmonates (JAs) in plant responses to abiotic stresses, particularly drought [2]. Despite growing evidence linking JAs to drought responses [31], the precise effects of JAs remain unclear, largely due to the variability in experimental conditions and plant material studied. Furthermore, most studies have focused primarily on drought responses in leaves with limited attention given to roots [27,32] or to coordinated analyses of both organs. In this context, our findings reveal distinct patterns of JA behaviour in roots compared to leaves. Under drought conditions, JA tended to accumulate in leaves, whereas a consistent reduction was observed in roots, with differences between drought-tolerant and susceptible genotypes as well as among individual jasmonate species. Although the dynamics in roots under drought were not strictly linear, both JA and JA-Ile showed transient increases followed by a decline, with overall levels remaining markedly lower than in well-watered controls. Notably, the induction of JA and JA-Ile in leaves and their concomitant reduction in roots occurred from moderate to severe drought stress, whereas changes in OPDA content occurred at earlier stages of water depletion. These genotype-dependent patterns highlight the contrasting jasmonate dynamics operating in leaves and roots during progressive drought.

In addition to drought-induced changes, temporal fluctuations in jasmonate levels were also detected in well-watered control plants, likely reflecting ongoing developmental processes. Jasmonates are known regulators of both root and shoot development, and their levels can vary across different stages of plant growth [41,42]. Such fluctuations probably mirror the coordination between JA signalling and processes such as root elongation, branching, and tissue differentiation. This constitutive variability underscores the need to interpret drought-induced responses relative to genotype-specific baseline levels, rather than absolute changes alone.

Changes in JAs levels in leaves during drought might be closely linked to transpiration regulation, given that jasmonates have been shown to regulate stomatal closure and transpiration water loss [43,44]. Following a slow and gradual water depletion, previous studies in this oat system have shown that drought resistance in both Flega and Patones is not associated with a tight and rapid stomatal closure. Instead, tolerance relies on a finely tuned modulation of stomatal behaviour that limits reactive oxygen species accumulation while minimizing negative effects on photosynthesis [36,38,40]. Within this context, the modest increase in OPDA observed in leaves, together with elevated JA and JA-Ile levels, may contribute to this fine-tuned stomatal adjustment. However, the contrasting effects of exogenous OPDA and JA on drought symptoms suggest that the regulation of leaf responses involves a complex and hormone-specific balance, underscoring the need for further studies to disentangle the precise physiological roles of individual JAs and their interactions with other hormones in leaves.

Our results differ from those of De Domenico et al. [32], who reported a coordinated induction of jasmonate biosynthetic genes accompanied by increased JA, JA-Ile and OPDA levels in chickpea roots subjected to drought. These contrasting outcomes may stem from differences in the experimental imposition of water stress. While De Domenico et al. [31] transplanted four-week-old plants directly into dry soil, drought in the present study was applied through gradual water depletion (Figure S1), with plants not reaching the wilting point even after 18 days. Additionally, species-specific physiological and hormonal differences between oat and chickpea may contribute to these divergent JAs responses. In our study, the overall reduction in OPDA and other JAs in drought-stressed roots was supported by the significant downregulation of the 13-LOX gene. Given that 13-LOX catalyzes the initial and rate-limiting step of the octadecanoid pathway [45], its repression would be expected to constrain downstream JA production. By contrast, the accumulation of OPDA observed in Flega roots relative to the tolerant, Patones genotype appears to be associated with strong induction of AOS expression. Since the AOS promoter in *Arabidopsis* is responsive to OPDA, JA, or salicylic acid [45,46], it is likely that AOS exerts a major control over the entire pathway. Thus, the differential AOS expression in our system would explain the distinct patterns of OPDA accumulation observed in Flega and Patones under drought.

Furthermore, in Flega, the stronger induction of AOS expression suggests an increased flux toward formation of 13-hydroperoxyoctadecatrienoic acid (13-HPOT) into the allene oxide intermediate. However, AOC transcript levels in Flega were not proportionately increased, potentially creating a bottleneck in this step of the pathway [47]. Under aqueous conditions, the unstable allene oxide intermediate can spontaneously cyclize to form OPDA enantiomers [3], underscoring the need for a balanced AOS-AOC system [4]. An imbalance, combined with possible post-transcriptional regulation of OPR3, may contribute to limiting OPDA conversion to JA and could help explain the relatively higher OPDA levels observed in Flega [15]. In contrast, the drought-tolerant Patones genotype might maintain a more balanced metabolic flux through these early steps of the pathway, consistent with its lower OPDA levels under drought. Overall, these patterns are consistent with upstream regulation of the JA pathway contributing to the contrasting OPDA dynamics observed between genotypes, although additional studies will be needed to establish the underlying mechanisms.

A key observation from our study was that the changes in root OPDA content during drought differed markedly from those of JA and JA-Ile. Although both cultivars showed reduced OPDA content in roots under drought, the timing and magnitude of these reductions clearly distinguished the drought-tolerant genotype Patones from the susceptible Flega. The kinetics of the response, rather than absolute OPDA levels, seem to differentiate the tolerant genotype, consistent with dynamic hormonal regulation reported for other stress-related metabolites [2,15,48]. To further assess the functional relevance of OPDA in root-mediated drought responses, OPDA was supplied to the root neck. This treatment exacerbated visual drought symptoms in both cultivars, reducing leaf relative water content, without affecting transpiration rates. This suggests that OPDA might be inhibiting root formation and impairing the development of new and thin roots, thereby diminishing root hydraulic conductance and compromising leaf water status. Previous studies have reported jasmonate-mediated inhibition of root growth [49] and OPDA-induced reductions in root development [50]. Given that fine roots represent the most active portion of the root system for water uptake [50,51,52,53], the reduction in fine-root length observed after OPDA application is consistent with impaired water acquisition under drought.

Interestingly, while exogenous OPDA reduced root growth, JA application did not affect root development. JA treatment ameliorated drought symptoms in leaves, which aligns with the increase in leaf JA observed in Patones under high-to-severe drought. Although JA has been reported to inhibit root growth under some conditions [54], our results did not show this effect, possibly reflecting differences in concentration, timing, or mode of hormone delivery. Alternatively, drought-stressed plants might activate compensatory mechanisms to mitigate JA’s inhibitory effects. Importantly, the effects observed after OPDA treatment on the root traits measured were more pronounced than those elicited by JA, suggesting that the OPDA-related response is not solely due to its downstream conversion to JA. Recent findings in wheat support this interpretation, where loss-of-function mutations in OPRIII, an enzyme involved in OPDA conversion, resulted in longer seminal roots, while increased OPRIII dosage or overexpression led to reduced seminal root growth and enhanced JA/JA-Ile accumulation [12]. These parallels suggest that OPDA levels and their conversion to JA may influence specific root traits associated with root elongation. It is important to note that hormone measurements after OPDA application were performed at 3 days, a time point chosen to capture sustained systemic effects under drought. Therefore, an earlier transient increase in endogenous OPDA cannot be excluded, and our data reflect later stages of OPDA turnover rather than its initial peak.

Beyond the phenotypic effects observed in our study, several recent advances provide plausible molecular frameworks through which OPDA may influence specific root traits. OPDA has emerged as an autonomous signalling molecule with activities that are partially independent of JA-Ile, triggering transcriptional responses distinct from canonical COI1-JAZ-mediated signalling [55]. In *Arabidopsis*, OPDA can activate COI1-independent gene networks involving TGA transcription factors and electrophilic oxylipin signalling, thereby modulating redox-regulated processes that may affect cell division or elongation in root tissues [56]. More recently, cyclophilin 20-3 has been identified as an OPDA-binding protein that redirects reducing power from thioredoxin F2 toward sulfur-assimilation pathways, generating redox signals capable of driving nuclear transcriptional reprogramming [57]. Additionally, OPDA-triggered glutathione signalling and S-glutathionylation events have been proposed to regulate redox-sensitive enzymes and retrograde signalling pathways, integrating stress cues with growth adjustments [58]. Although these mechanisms have not yet been studied in oat, they provide a conceptual framework in which OPDA could influence fine-root traits under drought through redox-dependent signalling, COI1-independent transcriptional regulation, or metabolic feedbacks in the jasmonate pathway.

Strikingly, we observed a rapid transformation of OPDA into JA following its injection, a process that aligns with the general regulation of JAs biosynthesis. However, this rapid conversion does not diminish the functional relevance of OPDA. On the contrary, the requirement for tight regulation of OPDA levels may reflect its specific signalling properties, particularly under stress conditions. Given that JA typically acts at higher concentrations, the plant may actively convert excess OPDA into JA to prevent its overaccumulation. Similar metabolic control occurs in other hormone pathways, such as auxin and gibberellin [59,60]. Previous work has highlighted distinct biological roles for OPDA and JA in stress responses [61], supporting the possibility that OPDA contributes to drought responses through mechanisms partially independent of JA-mediated regulation. In this context, several OPDA-responsive pathways described in *Arabidopsis* provide plausible mechanistic links to the effects observed here, including COI1-independent transcriptional responses mediated by TGA factors, modulation of reactive electrophile species, and interactions with auxin transport or cell-cycle regulation. Although these pathways have not yet been characterized in oat, they represent plausible mechanisms through which OPDA could influence fine-root traits under drought and therefore warrant future investigation.

## 4. Materials and Methods

### 4.1. Plant Material, Growth Condition and Sampling

All experiments were carried out with the oat cultivars (cvs) Flega and Patones, which are susceptible and tolerant to drought stress, respectively [35,36,38]. Patones was developed by ‘Instituto Madrileño de Investigación y Desarrollo Rural, Agrario y Alimentario’ (IMIDRA, Madrid, Spain), and ‘Plant Genetic Resources Center’ (INIA, Madrid, Spain) provided the seeds. Flega was developed by the Cereal Institute (Thermi-Thessaloniki, Greece). Genetic analyses of these cultivars show that they are not closely related [62].

Seedlings were grown in 0.75 L pots filled with peat:sand (3:1) in a growth chamber at 20 °C, 65% relative humidity and under 12 h dark/12 h light periods with 250 μmol m^−2^ s^−1^ photon flux density supplied by white fluorescent tubes (OSRAM). During growth, trays carrying the pots were watered regularly. To impose drought, when plants had two developed leaves and the third unrolled, watering was withheld [35,36,38] for a period of 18 days, resulting in a gradual depletion of soil water. Control plants were watered as described above throughout the whole experiment. During the drought treatment, the relative soil water content (RWC) was monitored daily, reaching a level of approximately 15–20% by day 18, which is consistent with previous drought-related studies on oat [28]. This allowed Flega and Patones plants to be subjected to similar soil relative water content during the whole drought time course [35,36,38].

Sampling times were chosen to cover different levels of RWC: still-sufficient water (approximately 6 days after water withholding (daww), 55–60% sRWC), mild water deficit (9 daww, 40–45% sRWC), moderate water deficit (12 daww, 30–35% sRWC), high water deficit (15 daww, 20–25% sRWC) and severe water deficit (18 daww; 15–20% sRWC). At the latest time point, plants were 38 days-old and the droughted plants had not reached the wilting point.

### 4.2. Visual Assessment of Drought Symptoms

Drought symptoms were scored in ten replicates per genotype/treatment as described by [35,36,38]. Briefly, drought severity values were assessed daily according to a 0–5 scale where 0 = vigorous plant, with no leaves showing drought symptoms; 1 = one or two leaves show slight drought symptoms (less turgor) but most leaves remain erect; 2 = most leaves show slight levels of drought stress, but one or two leaves still show no drought symptoms; 3 = all leaves show drought symptoms but these are not severe; 4 = all leaves show severe drought symptoms including incipient wilting; 5 = the whole plant is wilted with all leaves starting to dry, appearing rolled and/or shrunken (for pictures see [36]). These data were used to calculate the area under the drought progress curve (AUDPC) similarly to the area under the disease progress curve widely used in disease screenings [39] using the formula:

AUDPC = ∑ki = 1 ½ [(Si + Si + 1)(ti + 1 − ti)]

where Si is the drought severity at assessment date i, ti is the number of days after the first observation on assessment date i and k is the number of successive observations.

### 4.3. Jasmonate Quantification

At each sampling time, six plants per genotype and treatment were harvested at each time point and pooled in groups of two, yielding three biological replicates. Roots were washed under tap water to remove soil residues, and leaves and roots were frozen in liquid nitrogen and stored at −80 °C until assessment. Each sample consisted of a pool of two complete root systems or leaves from independent plants. JAs from samples were extracted and quantified as previously described [63] with slight modifications. Briefly, 0.2 g of dry plant material was extracted in 2 mL of distilled H_2_O after spiking with 100 ng dihydrojasmonic acid, as an internal standard. After centrifugation (10,000× *g* at 4 °C), supernatants were recovered and pH adjusted to 3.0 with 30% acetic acid. The acidified water extract was partitioned twice against 3 mL of diethyl ether. The organic layer was recovered and evaporated under vacuum in a centrifuge concentrator (Speed Vac, Jouan, Saint Herblain Cedex, France). The dry residue was then resuspended in a 9:1 H_2_O:MeOH solution by sonication. The resulting solution was filtered and directly injected into a UPLC system (Waters ACQUITY UPLC SDS, Waters Corporation Milford, MA, USA) interfaced to a TQD triple quadrupole (Micromass Ltd., Manchester, UK) mass spectrometer through an orthogonal Z-spray electrospray ion source. Separations were carried out on a Gravity C18 column (50 mm × 2.1 mm, 1.8-μm, Macherey-Nagel GmbH, Düren, Germany) using a linear gradient of MeOH and H_2_O supplemented with 0.1% acetic acid at a flow rate of 300 μL min^−1^. Transitions for JA/DHJA (209 > 59/211 > 59), OPDA (291 > 165), and JA-Ile (322 > 130) were monitored in negative ionization mode. Quantitation was achieved by external calibration with known amounts of pure standards using Masslynx v4.1 software (Micromass Ltd., Manchester, UK).

### 4.4. RNA Extraction and cDNA Amplification

Total RNA was extracted from 100 mg of ground tissue of three plants per genotype and treatment (control or 15 daww) using previously reported protocols [64,65]. RNA was purified using the RNeasy^®^ Minelute Cleanup Kit (QIAGEN, Hilden, Germany). Removal of any residual genomic DNA in RNA samples was verified by PCR amplification from total RNA (with no cDNA synthesis step) using the designed primers listed in Table S1. RNA samples containing DNA were further DNase treated until no PCR amplification of RNA samples was obtained. Prior to retro-transcription the concentration and integrity of RNA were verified by measuring the optical density at 260 nm and the OD260/OD280 absorption ratio with a NanoDrop ND-1000 spectrophotometer (Thermo Scientific, Wilmington, DE, USA). First- and second-strand of complementary DNA (cDNA) were synthesized using SuperScript^®^ III First-Strand (Invitrogen, Carlsbad, CA, USA) and DNA Polymerase I (New England BioLabs, Ipswich, MA, USA), respectively. cDNA samples were cleaned by the QIAquick PCR Purification Kit (QIAGEN, Hilden, Germany) and DNase treated by the RNase-Free DNase Set (QIAGEN, Hilden, Germany), according to the manufacturer’s recommendations. Conventional RT-PCR and PCR assays followed by gel electrophoresis were performed to verify the amplification of cDNA using the designed primers. The quality and quantity of cDNA were determined by running aliquots in agarose gels and by spectrophotometric analysis in a NanoDrop ND-1000 spectrophotometer (Thermo Scientific, Wilmington, DE, USA).

### 4.5. Gene Expression Analysis by Real-Time RT-PCR

Relative expression of oat genes involved in the jasmonate biosynthesis pathway was quantified 15 daww (20–25% soilRWC) using the primers (Table S1), designed with the Universal Probe Library Assay Design Center (Roche). At the time of primer design, the oat reference genome was not available; therefore, primers were developed from oat transcripts retrieved by BLAST 2.7 searches against *Arabidopsis* gene sequences in the NCBI TSA database (NCBI, last accessed January 2026). For genes with multiple isoforms, we selected those previously associated with drought or stress responses. After the release of the oat reference genome (OTpepsico_v2; [66]), we confirmed that our primers amplify the main annotated homologs for each enzyme: 13-LOX (2Dg0001160 and 6A6Ag0000269, encoding LOX2.3), AOS1 (7Cg0000672, 4Ag0001616, and 4Dg0001965, encoding AOS1), AOC (4Dg0000401 and 4Ag0000059, encoding AOC), OPR (7Ag0001092, encoding OPR1), ACX (7Ag0000538 and 5Cg0003403, encoding ACX1), MYC (1Cg0001681, 1Dg0000974, and 1Ag0000985, encoding MYC2), JAR (1Ag0003670, 1Ag0003668, 1Dg0002189 and 1Dg0002189 encoding JAR1), and COI (4Dg0002617, 4Ag0003065, and 3Cg0002898) encoding COI1. This analysis confirms that our primers target the main functional homologs within the JA biosynthetic and signalling pathways. GADPH was used as the internal control [67]. Real-time qRT-PCR was conducted using the StepOne Real-Time PCR System (Applied Biosystems, Foster City, CA, USA) and FastStart Universal SYBR Green Master (Rox) (Roche, Basel, Switzerland), following the manufacturer’s recommendations. Each reaction was performed on cDNA obtained from at least 3 independent biological replicates, with 2 technical qPCR replicates per sample. Gene expression was analyzed for each of the JA/JA-Ile biosynthetic genes, as well as for the reference gene GADPH. The reaction mixture contained 10 μL of SYBR Green master mix, 6 μL of each primer set (Table S1) and 65 ng of cDNA or standard solution as template. The amplification conditions were 95 °C for 10 min, followed by 40 cycles of amplification at 95 °C for 15 s and 60 °C for 1 min. Following amplification, a melting curve program 95 °C for 15 s, 60 °C for 1 min and 60 to 95 °C at a heating rate of 0.3 °C/min was used. To ensure amplification specificity, melting curve analysis consistently revealed a single peak for each gene, confirming the amplification of unique products. The fold changes in JA-associated gene transcripts in different treatments versus control (i.e., well-watered plants) were normalized using the Ct and efficiency obtained for the GADPH amplification run on the same cDNA templates according to the 2^−∆∆Ct^ method.

### 4.6. Exogenous Application of OPDA and JA

To assess the effects of OPDA and JA on drought responses in oats, 25 µL of a 30 µM solution were applied to ten and eight plants, respectively, per genotype and treatment by injection into the plant neck with a 2 mL fixed needle syringe (BD Discardit™ II, Becton Dickinson, Madrid, Spain) modified from [68]. This concentration was selected to remain within the range of physiologically relevant jasmonate levels observed in our material. A single application was performed when soil reached 50% RWC, approximately at 10 daww between 3 and 4 h after the onset of the light period. Control plants were treated similarly, with a solution of ethanol or methyl acetate at the same concentration, in which the hormones were dissolved. Plants were fixed in liquid nitrogen 3 days after the treatment and hormones were quantified in leaves and roots as stated above.

### 4.7. Leaf Relative Water Content

Leaf RWC was measured on ten plants per accession according to Barrs and Weatherley [69]. Measurements were carried out on the second leaves at 18 daww. Six hours after the onset of the light period, leaf blade segments were weighed (fresh weight; FW), using a three decimal precision balance (Kern PLJ model PLS 420-3F, Kern & Sohn GmbH, Balingen, Germany), floated on distilled water at 4 °C overnight and weighed again (turgid weight; TW). They were then dried at 80 °C for 48 h. After this, the dry weight (DW) was determined. RWC was then calculated as RWC = [(FW − DW)/(TW − DW)] × 100.

### 4.8. Transpiration

Transpiration was calculated on five replicates per genotype and treatment at 18 daww in two independent experiments. Pots were covered from both ends with polythene bags that were fixed to the pot with adhesive tape. A small slit was made in the top of the bag to allow the plant to pass through it. Control pots without plants showed minimal water loss. The initial and final (after 8 h in the central period of photoperiod) pot weight was taken, using a three decimal precision balance (Kern PLJ model PLS 420-3F, Kern & Sohn GmbH, Balingen, Germany), and water transpired was calculated by subtracting the final pot weight from the initial weight. Transpiration was normalized to the whole plant leaf area. The leaf area of each plant was measured after scanning the leaves using a commercial scanner (Epson Perfection V370 Photo, Epson, Suwa, Nagano, Japan) at a resolution of 600 pixels per mm, with ImageJ software (NIH, Bethesda, MD, USA; version 1.52) [70].

### 4.9. Morphological Root Trait Assessment

Root harvesting and handling followed previously established procedures for minimizing mechanical damage [40]. Roots were harvested at 18 daww corresponding to a soil RWC of 15–20%, using five roots per genotype and treatment. To minimize root damage, the peat substrate, which was sifted prior to sowing to remove coconut fibre, was first carefully loosened by immersing the roots in a bucket of water. The soil was then gently removed by hand to reduce mechanical stress. Following this, the roots were lightly showered with water on a sieve lined with filter paper, which efficiently removed any remaining soil without damaging the roots, preserving both the overall root system and fine roots (diameter between 0 and 40 μm). Subsequently, roots were fixed in 70% ethanol and stained with an abundant volume of 0.01% neutral red (Sigma Chemical Co., St. Louis, MO, USA) for 24 h to increase contrast in the image staining solution [71]. The stained roots were placed in a transparent tray with a thin layer of water and scanned using a scanner (Epson Perfection V370 Photo, Epson, Suwa, Nagano, Japan) at a resolution of 600 pixels per mm. Root images were analyzed using WinRHIZO (Regent Instruments Inc., Québec City, QC, Canada) as described by [72] and different root morphological parameters were measured including total root length and fine roots.

### 4.10. Statistical Analysis

For statistical analysis, data recorded as percentages were transformed to arcsine square roots (transformed value = 180/п × arcsine [√(%/100)]) to normalize data and stabilize variances throughout the data range. However, for ease of understanding, means of raw percentage data are presented in figures. Transformed data were subjected to ANOVA using SPSS software (IBM Corp., Armonk, NY, USA; version 26.0) and residual plots were inspected to confirm normality of the distribution. Significance of differences between means was determined by contrast analysis (Scheffe’s).

## 5. Conclusions

Our data shows that the drought-resistant oat cultivar significantly increased the leaf level of both JA and JA-Ile, whereas a reduction in JAs levels was observed in the roots of both the resistant and susceptible cultivars. Notably, the profiles of OPDA content in roots during gradual water depletion were distinct from those observed for JA or JA-Ile. Both cultivars experienced a reduction in OPDA content in roots, supported by the significant downregulation of the 13-LOX gene. However, the resistant cultivar exhibited a significantly greater reduction in OPDA content compared with the susceptible cultivar. When OPDA was applied to the base of the root system, it exacerbated visual drought symptoms in both cultivars primarily by inhibiting root growth and reducing leaf relative water content. Taken together, these results suggest a differential role for JAs in drought tolerance, emphasizing not only the varied effects between different tissues but also the distinct roles of pathway intermediaries, highlighting a role for OPDA influencing fine root development to cope with drought.

## Data Availability

The raw data supporting the conclusions of this article will be made available by the authors on request.

## Supplementary Materials

The following supporting information can be downloaded at: https://www.mdpi.com/article/10.3390/plants15091312/s1. Table S1. Primers name and sequence, designed with the Universal Probe Library Assay Design Center (Roche), used for PCR amplification of ARN samples. Figure S1: Soil relative water content (sRWC) for Flega and Patones plants during the drought time course in different experiments; Figure S2: Quantification of jasmonic acid (JA), jasmonoyl-isoleucine (JA-Ile), 12-oxo-phytodienoic acid (OPDA), salicylic acid (SA), and abscisic acid (ABA) in oat leaves and roots of Flega and Patones after exogenous application of OPDA.

## Abbreviations

The following abbreviations are used in this manuscript:

OPDA	12-oxophytodienoic acid
JAs	Jasmonates
JA	Jasmonic acid
JA-Ile	Jasmonoyl-isoleucine

## References

[1] Bosch M., Wright L.P., Gershenzon J., Wasternack C., Hause B., Schaller A., Stintzi A. (2014). Jasmonic acid and its precursor 12-oxophytodienoic acid control different aspects of constitutive and induced herbivore defenses in tomato. Plant Physiol..

[2] Wasternack C., Hause B. (2013). Jasmonates: Biosynthesis, perception, signal transduction and action in plant stress response, growth and development. An update to the 2007 review in Annals of Botany. Ann. Bot..

[3] Brash A.R., Baertschi S.W., Ingram C.D., Harris T.M. (1988). Isolation and characterization of natural allene oxides: Unstable intermediates in the metabolism of lipid hydroperoxides. Proc. Natl. Acad. Sci. USA.

[4] Hofmann E., Zerbe P., Schaller A. (2006). The crystal structure of Arabidopsis allene oxide cyclase: Insights into the oxylipin cyclization reaction. Plant Cell.

[5] Staswick P.E., Tiryaki I., Rowe M.L. (2002). Jasmonate response locus JAR1 and several related *Arabidopsis* genes encode enzymes of the firefly luciferase superfamily that show activity on jasmonic, salicylic, and indole-3-acetic acids in an assay for adenylation. Plant Cell.

[6] Xu C., Zhang B., Du H., Di H., Zhang L., Dong L., Zeng X., Xing J., Zhang J., Li C. (2026). Jasmonate-mediated defense coordination in the cereal–pathogen arms race. New Crops.

[7] Hu S., Zhu X., Ding Y., Zhang J., Nie C., Chen Y. (2024). Jasmonic Acid Signaling in Environmental Stress Adaptation of Horticulture Crops. Adv. Plant Sci. Environ..

[8] Rehman M., Saeed M.S., Fan X., Salam A., Munir R., Yasin M.U., Khan A.R., Muhammad S., Ali B., Ali I. (2023). The multifaceted role of jasmonic acid in plant stress mitigation: An overview. Plants.

[9] Chen Q., Sun J., Zhai Q., Zhou W., Qi L., Xu L., Wang B., Chen R., Jiang H., Qi J. (2011). The basic helix-loop-helix transcription factor MYC2 directly represses PLETHORA expression during jasmonate-mediated modulation of the root stem cell niche in Arabidopsis. Plant Cell.

[10] Zhang N., Lv X., Yan Y., Meng Q., Wang C., Jing W., Zhang Y., Xiao Z., Zhang H. (2026). Physiological and molecular mechanisms of ethylene in sculpting rice root system. Agronomy.

[11] Jan M., Muhammad S., Jin W., Zhong W., Zhang S., Lin Y., Zhou Y., Liu J., Liu H., Munir R.M. (2024). Modulating root system architecture: Cross-talk between auxin and phytohormones. Front. Plant Sci..

[12] Gabay G., Wang H., Zhang J., Moriconi J.I., Burguener G.F., Gualano L.D., Howell T., Lukaszewski A., Staskawicz B., Cho M. (2023). Dosage differences in *12-OXOPHYTODIENOATE REDUCTASE* genes modulate wheat root growth. Nat. Commun..

[13] Pigolev A., Miroshnichenko D., Dolgov S., Savchenko T. (2021). Regulation of Sixth Seminal Root Formation by Jasmonate in *Triticum aestivum* L.. Plants.

[14] Goetz S., Hellwege A., Stenzel I., Kutter C., Hauptmann V., Forner S., McCaig B., Hause G., Miersch O., Wasternack C. (2012). Role of cis-12-oxo-phytodienoic acid in tomato embryo development. Plant Physiol..

[15] Dave A., Hernández M.L., He Z., Andriotis V.M., Vaistij F.E., Larson T.R., Graham I.A. (2011). 12-oxo-phytodienoic acid accumulation during seed development represses seed germination in Arabidopsis. Plant Cell.

[16] Stelmach B.A., Muller A., Hennig P., Laudert D., Andert L., Weiler E.W. (1998). Quantitation of the octadecanoid 12-oxo-phytodienoic acid, a signalling compound in plant mechanotransduction. Phytochemistry.

[17] Stotz H.U., Mueller S., Zoeller M., Mueller M.J., Berger S. (2013). TGA transcription factors and jasmonate-independent COI1 signalling regulate specific plant responses to reactive oxylipins. J. Exp. Bot..

[18] Fliegmann J., Schuler G., Boland W., Ebel J., Mithofer A. (2003). The role of octadecanoids and functional mimics in soybean defense responses. Biol. Chem..

[19] Monte I., Kneeshaw S., Franco-Zorrilla J.M., Chini A., Zamarreño A.M., García-Mina J.M., Solano R. (2020). An Ancient COI1-Independent Function for Reactive Electrophilic Oxylipins in Thermotolerance. Curr. Biol..

[20] Sheard L.B., Tan X., Mao H., Withers J., Ben-Nissan G., Hinds T.R., Kobayashi Y., Hsu F.-F., Sharon M., Browse J. (2010). Jasmonate perception by inositol-phosphate-potentiated COI1-JAZ co-receptor. Nature.

[21] Loreti E., Povero G., Novi G., Solfanelli C., Alpi A., Perata P. (2008). Gibberellins, jasmonate and abscisic acid modulate the sucrose-induced expression of anthocyanin biosynthetic genes in *Arabidopsis*. New Phytol..

[22] Suza W.P., Staswick P.E. (2008). The role of JAR1 in Jasmonoyl-L-isoleucine production during Arabidopsis wound response. Planta.

[23] Delfin J.C., Kanno Y., Seo M., Kitaoka N., Matsuura H., Tohge T., Shimizu T. (2022). AtGH3.10 is another jasmonic acid-amido synthetase in *Arabidopsis thaliana*. Plant J..

[24] Sanders P., Lee P., Biesgen C., Boone J., Beals T., Weiler E., Goldberg R. (2000). The arabidopsis *DELAYED DEHISCENCE1* gene encodes an enzyme in the jasmonic acid synthesis pathway. Plant Cell.

[25] Wasternack C., Strand M. (2018). Jasmonates: News on Occurrence, Biosynthesis, Metabolism and Action of an Ancient Group of Signaling Compounds. Int. J. Mol. Sci..

[26] Chini A., Monte I., Zamarreño A.M., Hamberg M., Lassueur S., Reymond P., Weiss S., Stintzi A., Schaller A., Porzel A. (2018). An opr3-independent pathway uses 4,5-didehydrojasmonate for jasmonate synthesis. Nat. Chem. Biol..

[27] De Ollas C., Arbona V., Gomez-Cadenas A., Dodd I.C. (2018). Attenuated accumulation of jasmonates modifies stomatal responses to water deficit. J. Exp. Bot..

[28] Kazan K. (2015). Diverse roles of jasmonates and ethylene in abiotic stress tolerance. Trends Plant Sci..

[29] Mujahid A., Muhammad H.M.D. (2024). Insights into Different Mitigation Approaches for Abiotic Stress in Horticultural Plants. Adv. Plant Sci. Environ..

[30] Kim E.H., Kim Y.S., Park S.-H., Koo Y.J., Do Choi Y., Chung Y.-Y., Lee I.-J., Kim J.-K. (2009). Methyl jasmonate reduces grain yield by mediating stress signals to alter spikelet development in rice. Plant Physiol..

[31] Riemann M., Dhakarey R., Hazman M., Miro B., Kohli A., Nick P. (2015). Exploring jasmonates in the hormonal network of drought and salinity responses. Front. Plant Sci..

[32] De Domenico S., Bonsegna S., Horres R., Pastor V., Taurino M., Poltronieri P., Imtiaz M., Kahl G., Flors V., Winter P. (2012). Transcriptomic analysis of oxylipin biosynthesis genes and chemical profiling reveal an early induction of jasmonates in chickpea roots under drought stress. Plant Physiol. Biochem..

[33] Gasperini D., Chételat A., Acosta I., Goossens J., Pauwels L., Goossens A., Dreos R., Alfonso E., Farmer E.E. (2015). Multilayered Organization of Jasmonate Signalling in the Regulation of Root Growth. PLoS Genet..

[34] Sánchez-Romera B., Ruiz-Lozano J.M., Zamarreño A.M., García-Mina J.M., Aroca R. (2015). Arbuscular mycorrhizal symbiosis and methyl jasmonate avoid the inhibition of root hydraulic conductivity caused by drought. Mycorrhiza.

[35] Sánchez-Martín J., Canales F.J., Tweed J.K.S., Lee M.R.F., Rubiales D., Gomez-Cadenas A., Arbona V., Mur L.A.J., Prats E. (2018). Fatty acid profile changes during gradual soil water depletion in oats suggests a role for jasmonates in coping with drought. Front. Plant Sci..

[36] Sánchez-Martín J., Mur L.A.J., Rubiales D., Prats E. (2012). Targeting sources of drought tolerance within an *Avena* spp. collection through multivariate approaches. Planta.

[37] Canales F.J., Rispail N., García-Tejera O., Arbona V., Pérez-de-Luque A., Prats E. (2021). Drought resistance in oat involves ABA-mediated modulation of transpiration and root hydraulic conductivity. Environ. Exp. Bot..

[38] Sánchez-Martín J., Heald J., Kingston-Smith A., Winters A., Rubiales D., Sanz M., Mur L.A.J., Prats E. (2015). A metabolomic study in oats (*Avena sativa*) highlights a drought tolerance mechanism based upon salicylate signalling pathways and the modulation of carbon, antioxidant and photo-oxidative metabolism. Plant Cell Environ..

[39] Jeger M.J., Viljanen-Rollinson S.L.H. (2001). The use of the area under the disease-progress curve (AUDPC) to assess quantitative disease resistance in crop cultivars. Theor. Appl. Genet..

[40] Canales F.J., Nagel K.A., Muller C., Rispail N., Prats E. (2019). Deciphering Root Architectural Traits Involved to Cope with Water Deficit in Oat. Front. Plant Sci..

[41] Huang H., Liu B., Liu L., Song S. (2017). Jasmonate action in plant growth and development. J. Exp. Bot..

[42] Sharma M., Laxmi A. (2021). Jasmonates: A Thorough Insight into the Mechanism of Biosynthesis, Signaling and Action in Root Growth and Development. Rhizobiology: Molecular Physiology of Plant Roots.

[43] Tanaka Y., Sano T., Tamaoki M., Nakajima N., Kondo N., Hasezawa S. (2005). Ethylene inhibits abscisic acid-induced stomatal closure in Arabidopsis. Plant Physiol..

[44] Yin Y., Adachi Y., Nakamura Y., Munemasa S., Mori I.C., Murata Y. (2016). Involvement of OST1 protein kinase and PYR/PYL/RCAR receptors in methyl jasmonate-induced stomatal closure in arabidopsis guard cells. Plant Cell Physiol..

[45] Turner J.G., Ellis C., Devoto A. (2002). The jasmonate signal pathway. Plant Cell.

[46] Laudert D., Weiler E.W. (1998). Allene oxide synthase: A major control point in *Arabidopsis thaliana* octadecanoid signalling. Plant J..

[47] Stenzel I., Hause B., Miersch O., Kurz T., Maucher H., Weichert H., Ziegler J., Feussner I., Wasternack C. (2003). Jasmonate biosynthesis and the allene oxide cyclase family of *Arabidopsis thaliana*. Plant Mol. Biol..

[48] Vlot A.C., Dempsey D.A., Klessig D.F. (2009). Salicylic Acid, a multifaceted hormone to combat disease. Annu. Rev. Phytopathol..

[49] Staswick P.E., Su W.P., Howell S.H. (1992). Methyl jasmonate inhibition of root-growth and induction of a leaf protein are decreased in an *Arabidopsis thaliana* mutan. Proc. Natl. Acad. Sci. USA.

[50] Comas L.H., Becker S.R., Cruz V.M.V., Byrne P.F., Dierig D.A. (2013). Root traits contributing to plant productivity under drought. Front. Plant Sci..

[51] Rewald B., Ephrath J.E., Rachmilevitch S. (2011). A root is a root is a root? Water uptake rates of *Citrus* root orders. Plant Cell Environ..

[52] Shipley B., Meziane D. (2002). The balanced-growth hypothesis and the allometry of leaf and root biomass allocation. Funct. Ecol..

[53] Wasson A.P., Richards R.A., Chatrath R., Misra S.C., Prasad S.V.S., Rebetzke G.J., Kirkegaard J.A., Christopher J., Watt M. (2012). Traits and selection strategies to improve root systems and water uptake in water-limited wheat crops. J. Exp. Bot..

[54] Yan J., Zhang C., Gu M., Bai Z., Zhang W., Qi T., Cheng Z., Peng W., Luo H., Xie D. (2007). A downstream mediator in the growth repression limb of the jasmonate pathway. Plant Cell.

[55] Yi R., Li Y., Sha X. (2024). OPDA/dn-OPDA actions: Biosynthesis, metabolism, and signaling. Plant Cell Rep..

[56] Liu W., Park S.W. (2021). 12-oxo-Phytodienoic Acid: A Fuse and/or Switch of Plant Growth and Defense Responses?. Front. Plant Sci..

[57] Adhikari A., Kaur S., Forouhar F., Kale S., Park S.W. (2025). OPDA signaling channels resource (e-)allocation from the photosynthetic electron transfer chain to plastid cysteine biosynthesis in defense activation. J. Exp. Bot..

[58] Adhikari A., Park S.W. (2023). Reduced GSH Acts as a Metabolic Cue of OPDA Signaling in Coregulating Photosynthesis and Defense Activation under Stress. Plants.

[59] Yamaguchi S. (2008). Gibberellin Metabolism and Its Regulation. Annu. Rev. Plant Biol..

[60] Zhao Y. (2010). Auxin Biosynthesis and Its Role in Plant Development. Annu. Rev. Plant Biol..

[61] Taki N., Sasaki-Sekimoto Y., Obayashi T., Kikuta A., Kushiro M., Shimada Y., Ohta H. (2005). 12-oxo-phytodienoic acid triggers expression of a distinct set of genes and plays a role in wound-induced gene expression in Arabidopsis. Plant Physiol..

[62] Montilla-Bascón G., Sanchez-Martin J., Rispail N., Rubiales D., Mur L., Langdon T., Griffiths I., Howarth C., Prats E. (2013). Genetic diversity and population structure among oat cultivars and landraces. Plant Mol. Biol. Rep..

[63] De Ollas C., Hernando B., Arbona V., Gomez-Cadenas A. (2013). Jasmonic acid transient accumulation is needed for abscisic acid increase in citrus roots under drought stress conditions. Physiol. Plant..

[64] Chomczynski P., Sacchi N. (1987). Single-step method of RNA isolation by acid guanidinium thiocyanate-phenol-chloroform extraction. Anal. Biochem..

[65] Raeder U., Broda P. (1985). Rapid preparation of DNA from filamentous fungi. Appl. Microbiol..

[66] PepsiCo (2021). *Avena sativa*—OT3098 v2. https://wheat.pw.usda.gov/jb?data=/ggds/oat-ot3098v2-pepsico.

[67] Montilla-Bascon G., Rubiales D., Altabella T., Prats E. (2016). Free polyamine and polyamine regulation during pre-penetration and penetration resistance events in oat against crown rust (*Puccinia coronata* f. sp. *avenae*). Plant Pathol..

[68] Kondhare K.R., Hedden P., Kettlewell P.S., Farrell A.D., Monaghan J.M. (2014). Quantifying the impact of exogenous abscisic acid and gibberellins on pre-maturity alpha-amylase formation in developing wheat grains. Sci. Rep..

[69] Barrs H.D., Weatherley P.E. (1962). A re-examination of the relative turgidity technique for estimating water deficits in leaves. Aust. J. Biol. Sci..

[70] Schneider C.A., Rasband W.S., Eliceiri K.W. (2012). NIH Image to ImageJ: 25 years of image analysis. Nat. Methods.

[71] Schumacher T.E., Smucker A.J.M., Eshel A., Curry R.B. (1983). Measurement of short-term root-growth by prestaining with neutral red. Crop Sci..

[72] Himmelbauer M.L., Loiskandl W., Kastanek F. (2004). Estimating length, average diameter and surface area of roots using two different Image analyses systems. Plant Soil.

