# Supplementary material for: Differential Jasmonate Profiles in Oat Roots and Leaves Reveal a Role for 12-Oxo Phytodienoic Acid (OPDA) in Drought Tolerance by Modulating Root Growth
Source: Plants (Basel). 2026 Apr 24;15(9):1312. doi: 10.3390/plants15091312 (PMC13164625; doi:10.3390/plants15091312)
Supplement: Supplementary file 1 [file plants-15-01312-s001.zip › plants-4233915-supplementary.pdf]

plants MDPI

Supplementary Material

Differential jasmonate profiles in oat roots and leaves reveal a role for 12-Oxo Phytodienoic Acid in drought tolerance by modulating root growth.

Francisco J. Canales[1,2*], Gracia Montilla-Bascón[2], Nicolas Rispail[2], Vicent Arbona[3], Luis AJ Mur[1] and Elena Prats[2]

[1] Department of Life Sciences, Aberystwyth University UK
[2] CSIC, Institute for Sustainable Agriculture, Córdoba, Spain
[3] Ecofisiologia i Biotecnologia Dpt. Ciències Agràries i del Medi Natural. Universitat Jaume I - Campus Riu Sec. E-12071 Castelló de la Plana. Spain

* Correspondence: fjcanales@ias.csic.es

Table S1. Primers name and sequence, designed with the Universal Probe Library Assay Design Center (Roche), used for PCR amplification of ARN samples.

Name	Primer sequences
13-LOX	5′-GAGACATTCGGGCCAGTG-3′<br>5′-GAACATTTCCCTGTGGTGCT-3′
AOS	5′- CGTCGAAGGAGTCGTATCGT-3′<br>5′- AGGACTTCGTCGTGCTCATC-3′
AOC	5′- GGTCCTGACGGACACAGG-3′<br>5′- AAGAGCTTGTTCCAAGAACTCAC-3′
OPR	5′- CCGATTGAAGCACAGAACAG-3′<br>5′- ATGGTACAAGCGCTGTTGG-3′
ACX1	5′- ACAGGAAGTCGCCCAGGT-3′<br>5′- AGAACCTTTGCAACATTTACGC-3′
JAR1	5′- CCTTCAGGGAGATCCTGGTT-3′<br>5′- CGAGGCGTCTTGAACTGG-3′
COI1	5′- AGCTGTCAGGCCTAACTGAGA-3′<br>5′- GGATGATGATCCAGGCCTAC-3′
MYC2	5′- CTGGCAAAAACACAACAGGA-3′<br>5′- ATGATAGTTAGCAGCAGCTGGA-3′
GADPH	5′-GCTCAAGGGTATCATGGGTTACG-3′<br>5′-GCAATTCCACCCTTAGCATACAAG-3′

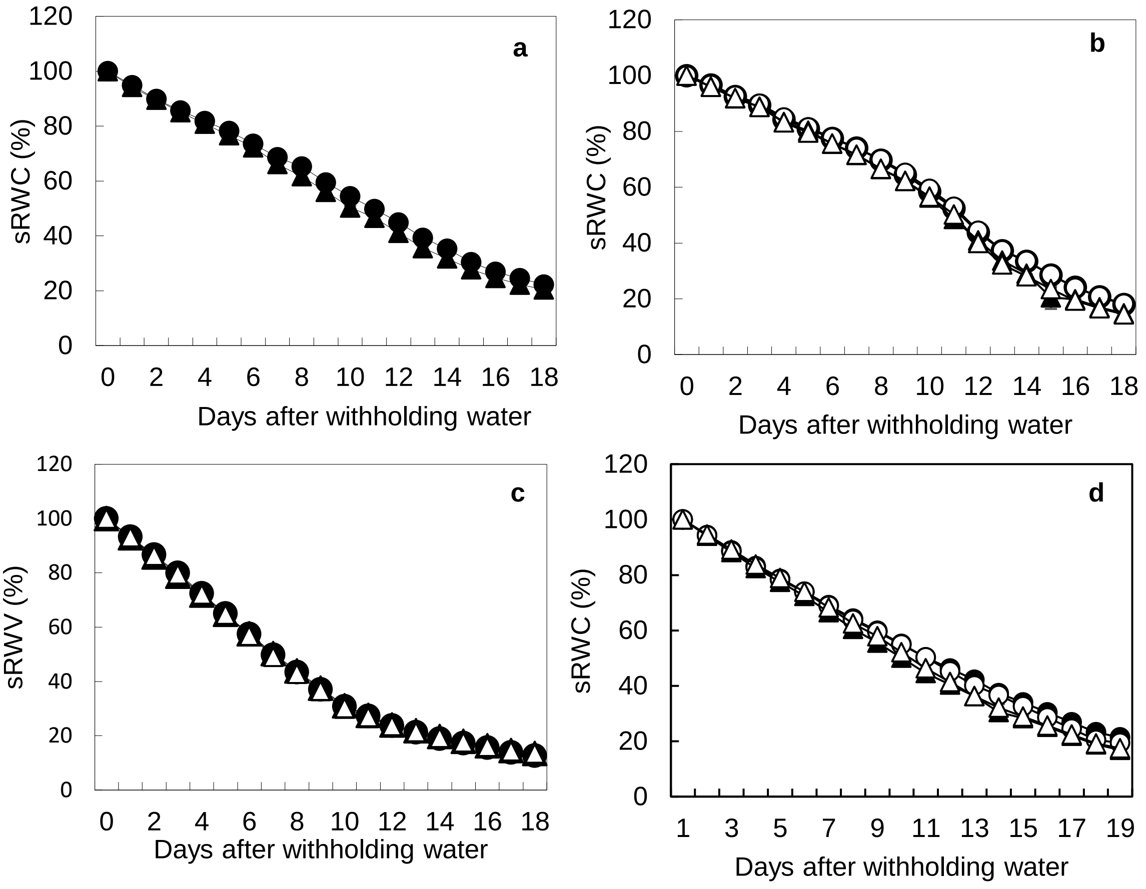

Figure S1. Soil relative water content (sRWC) for Flega (triangles) and Patones (circles) plants during the drought time course in different experiments: (a) plants not subjected to OPDA or JA treatment, used for hormonal level measurements; (b) plants treated with OPDA (open symbols) or mock-treated (solid symbols) for hormonal level measurements;(c) plants treated with OPDA (open symbols) or mock-treated (solid symbols) for physiological, root morphology, and gene expression analyses; and (d) plants treated with JA (open symbols) or mock-treated (solid symbols) for physiological and root morphology analyses. Data represent means of at least ten replicates ± standard error.

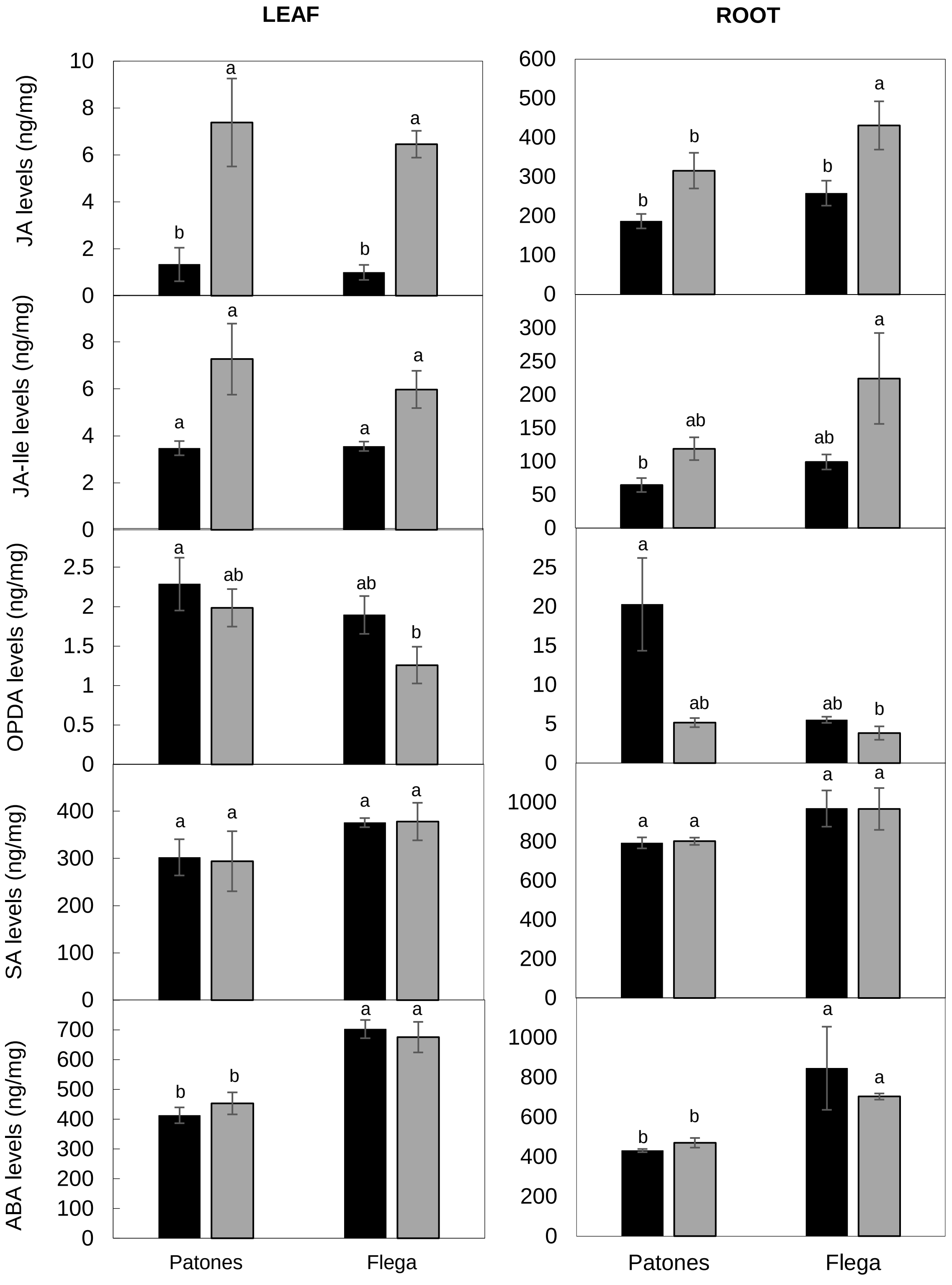

Figure S2. Quantification of jasmonic acid (JA), jasmonoyl-isoleucine (JA-Ile), 12-oxo-phytodienoic acid (OPDA), salicylic acid (SA), and abscisic acid (ABA) in oat leaves and roots of Flega and Patones after exogenous application of OPDA. Grey bars represent OPDA-treated plants under drought conditions; black bars represent mock-treated with ethanol plants under drought. Drought-stressed control plants received the same ethanol treatment as OPDA-treated plants but without OPDA. Each set comprised at least six biological replicates (different plants) per oat genotype and treatment. Sampled tissues from 2 different plants (2 technical replicates) were pooled making one biological replicate, so data in the figure are mean of the three biological replicates ± standard errors. Different letters indicate for each panel significant differences at $p < 0.05$ according to Scheffé's post hoc test.
